# Pyrazine­diium bis­(3-carb­oxy-4-hydroxy­benzene­sulfonate) dihydrate

**DOI:** 10.1107/S1600536808017777

**Published:** 2008-06-19

**Authors:** Jia-Rong Wang, Ze-Hui Yang, Chun-Hai Liu, Ling-Lan Li

**Affiliations:** a College of Chemical Engineering, Ningbo University of Technology, Ningbo, Zhejiang 315016, People’s Republic of China; b Hunan Jiuzhitang Co. Ltd, Changsha, Hunan 410008, People’s Republic of China

## Abstract

Pyrazine and 5-sulfosalicylic acid crystallize from a methanol solution containing water as the title salt, C_4_H_6_N_2_^2+^·2C_7_H_5_O_6_S^−^·2H_2_O. The pyrazine­diium cation sits on an inversion center. The component ions and water mol­ecules are linked by inter­molecular O—H⋯O, N—H⋯O and C—H⋯O hydrogen bonds into layers running parallel to the (10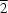) plane.

## Related literature

For related literature, see: Smith *et al.* (2004 ▶, 2005 ▶); Meng *et al.* (2008 ▶).
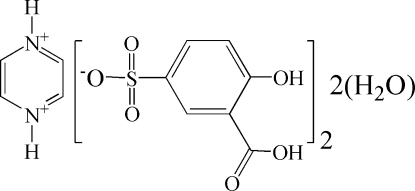


## Experimental

### 

#### Crystal data

C_4_H_6_N_2_^2+^·2C_7_H_5_O_6_S^−^·2H_2_O
*M* *_r_* = 552.48
Triclinic, 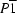

*a* = 6.7887 (5) Å
*b* = 6.9256 (6) Å
*c* = 13.0349 (10) Å
α = 100.663 (7)°
β = 97.761 (9)°
γ = 107.735 (9)°
*V* = 561.52 (9) Å^3^
*Z* = 1
Mo *K*α radiation
μ = 0.32 mm^−1^
*T* = 298 (2) K
0.20 × 0.10 × 0.08 mm

#### Data collection

Bruker SMART APEX CCD area-detector diffractometer
Absorption correction: multi-scan (*SADABS*; Sheldrick, 1997 ▶) *T*_min_ = 0.929, *T*_max_ = 0.975
5286 measured reflections
2412 independent reflections
1977 reflections with *I* > 2σ(*I*)
*R*_int_ = 0.064

#### Refinement

*R*[*F* ^2^ > 2σ(*F* ^2^)] = 0.045
*wR*(*F* ^2^) = 0.131
*S* = 1.08
2412 reflections
178 parameters
H atoms treated by a mixture of independent and constrained refinement
Δρ_max_ = 0.32 e Å^−3^
Δρ_min_ = −0.51 e Å^−3^

### 

Data collection: *SMART* (Bruker, 2001 ▶); cell refinement: *SMART*; data reduction: *SAINT-Plus* (Bruker, 2001 ▶; program(s) used to solve structure: *SHELXS97* (Sheldrick, 2008 ▶); program(s) used to refine structure: *SHELXL97* (Sheldrick, 2008 ▶); molecular graphics: *PLATON* (Spek, 2003 ▶); software used to prepare material for publication: *PLATON*.

## Supplementary Material

Crystal structure: contains datablocks global, I. DOI: 10.1107/S1600536808017777/cs2079sup1.cif

Structure factors: contains datablocks I. DOI: 10.1107/S1600536808017777/cs2079Isup2.hkl

Additional supplementary materials:  crystallographic information; 3D view; checkCIF report

## Figures and Tables

**Table 1:** Hydrogen-bond geometry (Å, °)

*D*—H⋯*A*	*D*—H	H⋯*A*	*D*⋯*A*	*D*—H⋯*A*
C8—H8⋯O6	0.93	2.40	3.273 (3)	156
C9—H9⋯O6^i^	0.93	2.41	3.240 (3)	149
C6—H6⋯O1^ii^	0.93	2.49	3.249 (2)	139
N1—H1*B*⋯O7	0.87 (3)	1.71 (3)	2.580 (3)	176 (3)
O7—H7*A*⋯O5	0.83 (3)	1.84 (3)	2.659 (2)	171 (3)
O7—H7*B*⋯O4^i^	0.83 (3)	1.81 (3)	2.631 (3)	172 (3)
O3—H3*A*⋯O2	0.83 (3)	1.92 (3)	2.625 (2)	143 (3)
O1—H1*A*⋯O5^ii^	0.82 (3)	1.92 (3)	2.719 (2)	163 (3)

Symmetry codes: (i) 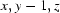; (ii) 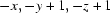.

**Fig. 1.**
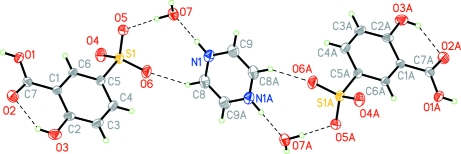
Molecular structure of the title salt showing the atom-numbering scheme. Displacement ellipsoids are drawn at the 50% probability level. H-bonds are shown in dashed lines. Symmetry code: (A) 2 - x, 1 - y, 2 - z.

**Fig. 2.**
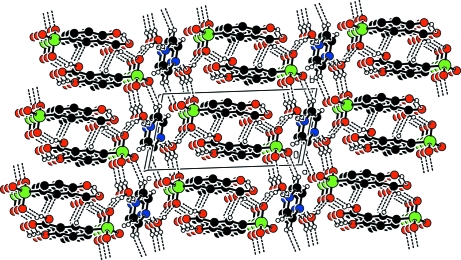
Excerpt from the crystal structure showing the formation of the two-dimensional layers running parallel to the [102] direction. Hydrogen bonds are shown as dashed lines. For the sake of clarity, H atoms not involved in the motif have been omitted from the drawing.

## supplementary crystallographic information

### Comment

5-sulfosalicylic acid is strong organic acid (pKa = 2.85) which can release its sulfonic acid H to Lewis base N atoms, forming 1:1 molecular adducts in general (Smith *et al.*, 2004 and 2005). In order to gain more insight into these analogues, we have recently prepared an organic salt containing 5-H_2_SSA, whose crystal structure is reported here.

There are a half of a pyrazinediium cation, one each a 5-HSSA^- ^anion and a water molecule in the asymmetric unit. Similarly to analogous organic adducts reported by others (Meng *et al.*, 2008), the H atom is transferred from the sulfonic acid group to the pyrazine N atoms (Fig.1). Except for this, the other bond lengths and angles are usual.

By a combination of seven intermolecular hydrogen bonds (Table 1), these components are linked into a two-dimensional layers (Fig.2) running parallel to the (102) plane in the crystal.

Program *PLATON* (Spek, 2003) reports two distances indicating possible aromatic π-π stacking interactions between symmetry-center related benzene rings comprising atoms C1 to C6 in this crystal. Centroid distances between the closest symmetry-related benzene rings are 3.848 (2) and 4.239 (2) Å, respectively (symmetry codes: 1-x,1 -y, 1-z for the former one and 1-x, 2-y, 1-z for the latter one).

### Experimental

All the reagents and solvents were used as obtained without further purification. Equimolar amount of pyrazine (0.2 mmol, 16.0 mg) and 5-sulfosalicylic acid dihydrate (0.2 mmol, 50.8 g) were dissolved in 95% methanol (10 ml). The mixture was stirred for ten minutes at ambient temperature and then filtered. The resulting colorless solution was kept in air for two weeks. Plate shaped colorless crystals suitable for single-crystal X-ray diffraction analysis were grown by slow evaporation of the solvent.

### Refinement

H atoms bonded to C atoms were positioned geometrically with C–H = 0.93Å (aromatic) and refined in a riding mode [*U*_iso_(H) = 1.2*U*_eq_(aromatic C)]. H atoms bonded to N and O atoms were found in difference Fourier maps with N—H and O—H distances being refined freely [the refined distances are given in Table 1; *U*_iso_(H)= 1.2*U*_eq_(N) or 1.5*U*_eq_(O)].

### Crystal data

**Table:**

C_4_H_6_N_2_^2+^·2C_7_H_5_O_6_S^–^·2H_2_O	*Z* = 1
*M**_r_* = 552.48	*F*_000_ = 286
Triclinic, *P*1	*D*_x_ = 1.634 Mg m^−^^3^
Hall symbol: -P 1	Mo *K*α radiation λ = 0.71073 Å
*a* = 6.7887 (5) Å	Cell parameters from 2269 reflections
*b* = 6.9256 (6) Å	θ = 1.6–25.2º
*c* = 13.0349 (10) Å	µ = 0.32 mm^−^^1^
α = 100.663 (7)º	*T* = 298 (2) K
β = 97.761 (9)º	Plate, colourless
γ = 107.735 (9)º	0.20 × 0.10 × 0.08 mm
*V* = 561.52 (9) Å^3^	

### Data collection

**Table:**

Bruker SMART APEX CCD area-detector diffractometer	2412 independent reflections
Radiation source: fine focus sealed Siemens Mo tube	1977 reflections with *I* > 2σ(*I*)
Monochromator: graphite	*R*_int_ = 0.064
*T* = 298(2) K	θ_max_ = 27.0º
0.3° wide ω exposures scans	θ_min_ = 1.6º
Absorption correction: multi-scan(SADABS; Sheldrick, 1997)	*h* = −8→8
*T*_min_ = 0.929, *T*_max_ = 0.975	*k* = −5→8
5286 measured reflections	*l* = −16→16

### Refinement

**Table:**

Refinement on *F*^2^	Secondary atom site location: difference Fourier map
Least-squares matrix: full	Hydrogen site location: inferred from neighbouring sites
*R*[*F*^2^ > 2σ(*F*^2^)] = 0.045	H atoms treated by a mixture of independent and constrained refinement
*wR*(*F*^2^) = 0.131	*w* = 1/[σ^2^(*F*_o_^2^) + (0.0732*P*)^2^ + 0.0236*P*] where *P* = (*F*_o_^2^ + 2*F*_c_^2^)/3
*S* = 1.08	(Δ/σ)_max_ = 0.001
2412 reflections	Δρ_max_ = 0.32 e Å^−^^3^
178 parameters	Δρ_min_ = −0.51 e Å^−^^3^
Primary atom site location: structure-invariant direct methods	Extinction correction: none

### Fractional atomic coordinates and isotropic or equivalent isotropic displacement parameters (Å^2^)

**Table:**

	*x*	*y*	*z*	*U*_iso_*/*U*_eq_	
C1	0.4240 (3)	0.7285 (3)	0.48381 (14)	0.0311 (4)	
C2	0.6433 (3)	0.7939 (3)	0.48820 (17)	0.0361 (5)	
C3	0.7816 (3)	0.8439 (3)	0.58633 (18)	0.0439 (5)	
H3	0.9265	0.8851	0.5895	0.053*	
C4	0.7052 (3)	0.8328 (3)	0.67817 (17)	0.0425 (5)	
H4	0.7985	0.8671	0.7432	0.051*	
C5	0.4881 (3)	0.7704 (3)	0.67421 (15)	0.0338 (4)	
C6	0.3486 (3)	0.7183 (3)	0.57800 (14)	0.0323 (4)	
H6	0.2040	0.6762	0.5758	0.039*	
C7	0.2761 (3)	0.6708 (3)	0.38006 (15)	0.0362 (5)	
C8	0.9086 (4)	0.6243 (4)	0.96127 (19)	0.0543 (6)	
H8	0.8459	0.7119	0.9346	0.065*	
C9	0.8819 (4)	0.3018 (4)	0.99291 (18)	0.0511 (6)	
H9	0.8012	0.1641	0.9884	0.061*	
N1	0.7935 (3)	0.4275 (3)	0.95466 (13)	0.0466 (5)	
H1B	0.658 (4)	0.377 (4)	0.928 (2)	0.056*	
O1	0.0776 (2)	0.6158 (3)	0.38832 (11)	0.0484 (4)	
H1A	−0.005 (5)	0.578 (5)	0.331 (2)	0.073*	
O2	0.3328 (3)	0.6740 (3)	0.29528 (11)	0.0531 (4)	
O3	0.7255 (3)	0.8128 (3)	0.40112 (14)	0.0523 (4)	
H3A	0.633 (5)	0.788 (5)	0.348 (3)	0.078*	
O4	0.2517 (3)	0.8910 (3)	0.79365 (12)	0.0535 (4)	
O5	0.2546 (2)	0.5428 (2)	0.78053 (11)	0.0467 (4)	
O6	0.5615 (3)	0.8329 (3)	0.88036 (11)	0.0522 (4)	
O7	0.3909 (3)	0.2940 (3)	0.88232 (12)	0.0475 (4)	
H7A	0.346 (5)	0.362 (5)	0.844 (2)	0.071*	
H7B	0.340 (5)	0.169 (5)	0.849 (2)	0.071*	
S1	0.38501 (8)	0.76139 (8)	0.79128 (3)	0.0378 (2)	

### Atomic displacement parameters (Å^2^)

**Table:**

	*U*^11^	*U*^22^	*U*^33^	*U*^12^	*U*^13^	*U*^23^
C1	0.0308 (9)	0.0259 (10)	0.0391 (9)	0.0108 (8)	0.0074 (8)	0.0115 (8)
C2	0.0321 (10)	0.0272 (10)	0.0533 (11)	0.0111 (8)	0.0152 (8)	0.0130 (9)
C3	0.0247 (10)	0.0414 (12)	0.0655 (13)	0.0105 (9)	0.0075 (9)	0.0150 (10)
C4	0.0325 (10)	0.0412 (12)	0.0495 (11)	0.0106 (9)	−0.0022 (9)	0.0113 (9)
C5	0.0319 (10)	0.0293 (10)	0.0391 (9)	0.0103 (8)	0.0030 (8)	0.0090 (8)
C6	0.0265 (9)	0.0316 (11)	0.0391 (9)	0.0101 (8)	0.0044 (7)	0.0103 (8)
C7	0.0369 (11)	0.0349 (11)	0.0396 (10)	0.0125 (9)	0.0085 (8)	0.0145 (8)
C8	0.0648 (16)	0.0550 (16)	0.0518 (13)	0.0315 (13)	0.0084 (11)	0.0173 (11)
C9	0.0617 (15)	0.0454 (14)	0.0481 (12)	0.0214 (12)	0.0105 (11)	0.0113 (10)
N1	0.0443 (10)	0.0565 (13)	0.0383 (9)	0.0203 (10)	0.0045 (8)	0.0073 (9)
O1	0.0322 (8)	0.0695 (12)	0.0376 (7)	0.0083 (8)	0.0005 (6)	0.0184 (7)
O2	0.0521 (9)	0.0701 (12)	0.0390 (8)	0.0189 (9)	0.0136 (7)	0.0176 (7)
O3	0.0404 (9)	0.0604 (11)	0.0606 (10)	0.0151 (8)	0.0235 (7)	0.0190 (8)
O4	0.0572 (10)	0.0634 (11)	0.0488 (8)	0.0343 (9)	0.0092 (7)	0.0126 (7)
O5	0.0447 (9)	0.0477 (10)	0.0400 (7)	0.0053 (7)	0.0014 (6)	0.0148 (7)
O6	0.0515 (9)	0.0557 (10)	0.0383 (7)	0.0133 (8)	−0.0093 (7)	0.0064 (7)
O7	0.0571 (10)	0.0444 (10)	0.0467 (8)	0.0238 (8)	0.0082 (7)	0.0152 (7)
S1	0.0369 (3)	0.0405 (3)	0.0328 (3)	0.0128 (2)	−0.0006 (2)	0.0074 (2)

### Geometric parameters (Å, °)

**Table:**

C1—C6	1.398 (3)	C8—N1	1.329 (3)
C1—C2	1.408 (3)	C8—C9^i^	1.363 (4)
C1—C7	1.478 (2)	C8—H8	0.9300
C2—O3	1.341 (3)	C9—N1	1.329 (3)
C2—C3	1.397 (3)	C9—C8^i^	1.363 (4)
C3—C4	1.373 (3)	C9—H9	0.9300
C3—H3	0.9300	N1—H1B	0.87 (3)
C4—C5	1.395 (3)	O1—H1A	0.82 (3)
C4—H4	0.9300	O3—H3A	0.83 (3)
C5—C6	1.380 (2)	O4—S1	1.4556 (17)
C5—S1	1.766 (2)	O5—S1	1.4721 (15)
C6—H6	0.9300	O6—S1	1.4421 (14)
C7—O2	1.220 (2)	O7—H7A	0.83 (3)
C7—O1	1.310 (2)	O7—H7B	0.83 (3)
			
C6—C1—C2	119.40 (17)	O1—C7—C1	113.11 (16)
C6—C1—C7	120.76 (17)	N1—C8—C9^i^	120.2 (2)
C2—C1—C7	119.84 (17)	N1—C8—H8	119.9
O3—C2—C3	118.37 (18)	C9^i^—C8—H8	119.9
O3—C2—C1	122.21 (19)	N1—C9—C8^i^	119.6 (2)
C3—C2—C1	119.41 (18)	N1—C9—H9	120.2
C4—C3—C2	120.51 (19)	C8^i^—C9—H9	120.2
C4—C3—H3	119.7	C8—N1—C9	120.2 (2)
C2—C3—H3	119.7	C8—N1—H1B	122.3 (17)
C3—C4—C5	120.19 (19)	C9—N1—H1B	117.4 (18)
C3—C4—H4	119.9	C7—O1—H1A	113 (2)
C5—C4—H4	119.9	C2—O3—H3A	111 (2)
C6—C5—C4	120.25 (18)	H7A—O7—H7B	107 (3)
C6—C5—S1	118.43 (15)	O6—S1—O4	114.02 (10)
C4—C5—S1	121.30 (15)	O6—S1—O5	111.78 (9)
C5—C6—C1	120.23 (17)	O4—S1—O5	109.60 (10)
C5—C6—H6	119.9	O6—S1—C5	107.54 (10)
C1—C6—H6	119.9	O4—S1—C5	106.61 (9)
O2—C7—O1	123.31 (18)	O5—S1—C5	106.90 (9)
O2—C7—C1	123.58 (18)		
			
C6—C1—C2—O3	−177.99 (18)	C6—C1—C7—O2	−179.0 (2)
C7—C1—C2—O3	2.2 (3)	C2—C1—C7—O2	0.8 (3)
C6—C1—C2—C3	1.1 (3)	C6—C1—C7—O1	1.0 (3)
C7—C1—C2—C3	−178.71 (18)	C2—C1—C7—O1	−179.17 (18)
O3—C2—C3—C4	178.12 (19)	C9^i^—C8—N1—C9	0.2 (4)
C1—C2—C3—C4	−1.0 (3)	C8^i^—C9—N1—C8	−0.2 (4)
C2—C3—C4—C5	0.3 (3)	C6—C5—S1—O6	−176.94 (15)
C3—C4—C5—C6	0.3 (3)	C4—C5—S1—O6	1.7 (2)
C3—C4—C5—S1	−178.24 (16)	C6—C5—S1—O4	−54.28 (18)
C4—C5—C6—C1	−0.2 (3)	C4—C5—S1—O4	124.33 (19)
S1—C5—C6—C1	178.39 (14)	C6—C5—S1—O5	62.88 (17)
C2—C1—C6—C5	−0.5 (3)	C4—C5—S1—O5	−118.51 (18)
C7—C1—C6—C5	179.32 (17)		

Symmetry codes: (i) −*x*+2, −*y*+1, −*z*+2.

### Hydrogen-bond geometry (Å, °)

**Table:**

*D*—H···*A*	*D*—H	H···*A*	*D*···*A*	*D*—H···*A*
C8—H8···O6	0.93	2.40	3.273 (3)	156
C9—H9···O6^ii^	0.93	2.41	3.240 (3)	149
C6—H6···O1^iii^	0.93	2.49	3.249 (2)	139
N1—H1B···O7	0.87 (3)	1.71 (3)	2.580 (3)	176 (3)
O7—H7A···O5	0.83 (3)	1.84 (3)	2.659 (2)	171 (3)
O7—H7B···O4^ii^	0.83 (3)	1.81 (3)	2.631 (3)	172 (3)
O3—H3A···O2	0.83 (3)	1.92 (3)	2.625 (2)	143 (3)
O1—H1A···O5^iii^	0.82 (3)	1.92 (3)	2.719 (2)	163 (3)

Symmetry codes: (ii) *x*, *y*−1, *z*; (iii) −*x*, −*y*+1, −*z*+1.
